# Caffeine and Cognitive Functions in Sports: A Systematic Review and Meta-Analysis

**DOI:** 10.3390/nu13030868

**Published:** 2021-03-06

**Authors:** Jorge Lorenzo Calvo, Xueyin Fei, Raúl Domínguez, Helios Pareja-Galeano

**Affiliations:** 1 Sports Department, Facultad de Ciencias de la Actividad Física y del Deporte, Universidad Politécnica de Madrid, 28040 Madrid, Spain; jorge.lorenzo@upm.es; 2 Faculty of Sports Sciences, Universidad Europea de Madrid, 28670 Madrid, Spain; helios.pareja@gmail.com; 3 Studies Research Group in Neuromuscular Responses (GEPREN), University of Lavras, Lavras 37200-000, Brazil; raul_dominguez_herrera@hotmail.com; 4 Departamento de Motricidad Humana y Rendimiento Deporte, Universidad de Sevilla, 41013 Sevilla, Spain

**Keywords:** caffeine, cognitive function, ergogenic drinks, sport

## Abstract

Cognitive functions are essential in any form of exercise. Recently, interest has mounted in addressing the relationship between caffeine intake and cognitive performance during sports practice. This review examines this relationship through a structured search of the databases Medline/PubMed and Web of Science for relevant articles published in English from August 1999 to March 2020. The study followed PRISMA guidelines. Inclusion criteria were defined according to the PICOS model. The identified records reported on randomized cross-over studies in which caffeine intake (as drinks, capsules, energy bars, or gum) was compared to an identical placebo situation. There were no filters on participants’ training level, gender, or age. For the systematic review, 13 studies examining the impacts of caffeine on objective measures of cognitive performance or self-reported cognitive performance were selected. Five of these studies were also subjected to meta-analysis. After pooling data in the meta-analysis, the significant impacts of caffeine only emerged on attention, accuracy, and speed. The results of the 13 studies, nevertheless, suggest that the intake of a low/moderate dose of caffeine before and/or during exercise can improve self-reported energy, mood, and cognitive functions, such as attention; it may also improve simple reaction time, choice reaction time, memory, or fatigue, however, this may depend on the research protocols.

## 1. Introduction

Caffeine (1,3,7-trimethylxanthine) is among the supplements most commonly used by athletes of all sports modalities [1,2,3,4]. Since 2004, when caffeine was removed from the list of banned substances for sports, caffeine supplementation has reached a prevalence rate of 76% among international competition athletes [5]. In the 1970s, the first studies addressing the effect of caffeine on sports performance started to emerge. These studies identified an improvement in time to exhaustion in an endurance test, and such ergogenic effects were attributed to increased lipolysis and sparing of muscle glycogen [6]. Currently, the ergogenic capacity of caffeine is explained by its blocking effect on adenosine receptors [7] A_1_, A_2A_, and A_2B_ [8], due to the similar chemical structure of caffeine and adenosine. By blocking adenosine receptors at the neuromuscular level [9], caffeine enhances neuromuscular recruitment [10]. In addition, caffeine potentiates the Na^+^-K^+^ pump [11] and increases Ca^2+^ bioavailability at the myoplasm by inducing Ca^2+^ release from the sarcoplasmic reticulum and inhibition of its reuptake [12], resulting in the translocation of glycogen phosphorylase-b into isoform-a [13]. Furthermore, caffeine maximizes glycolytic activity through increased activity of the enzyme phosphofructokinase [14]. This means that, after ~60 min of caffeine supplementation coinciding with peak blood levels [15], caffeine has confirmed ergogenic effects in a wide range of sports activities [16,17]. These activities include as much of those involving a predominance of oxidative metabolism, such as endurance sport modalities [18,19], as those involving a predominance of non-oxidative metabolism, such as those requiring a high movement velocity [20] and power [21], i.e., the Wingate test [22], or a mixed metabolism, such as team sports [23], combat sports [24], or racquet sports [25].

So far, studies examining this topic have focused on the benefits of caffeine for physical performance, while its impacts on cognitive performance have received less attention. Cognitive functions include a broad range of basic mental operations, such as attention, memory, and executive functions involving working memory, decision-making, and multitasking, among others. An athlete’s attention, defined as the allocation of cognitive resources to internal or external stimuli, is key to successful sports performance [26]. The beneficial effects attributed to caffeine supplementation are based on the notion that, as adenosine inhibits the release of excitatory neurotransmitters, such as dopamine (excitatory neurotransmitter in the brain) [27], the antagonistic effects of caffeine could lead to the release of excitatory neurotransmitters (i.e., dopamine and noradrenaline) [28], and could thus exert central ergogenic effects [29]. Accordingly, caffeine supplementation is thought to promote a more favorable mood [30,31], increasing alertness and reducing the feeling of fatigue [32,33]. These effects could be considered beneficial for athletes practicing sports with high demands at both the physical and cognitive levels. For example, soccer is highly aerobic, but also includes a mix of anaerobic power and cognitive load, with all three contributing to a player’s performance and success [34,35,36]. In different studies, positive effects of caffeine have been reported in improving word learning speed and delayed recall [37], along with reaction time in response to tests, such as the Stroop test and the Rapid Visual Information Processing test, before and during exercise [38]. In addition, caffeine has been shown to improve reaction time, accuracy, and willingness to put physical effort into exercise [39].

Based on these potential ergogenic effects of caffeine supplementation on both physical and cognitive functions, this systematic review and meta-analysis sought to critically assess the effect of caffeine administered in the form of gum, capsules, drinks, or energy bars on several measures of cognitive performance in sports.

## 2. Materials and Methods 

### 2.1. Protocol

The systematic review and meta-analysis were conducted according to Preferred Reporting Items for Systematic Review and Meta-Analyses (PRISMA) guidelines [40]. To define the inclusion criteria, the PICOS model was followed [41] (see Table 1).

### 2.2. Search Strategy

The databases PubMed and Web of Science (WOS) were searched for articles published in English from 1 August 1999 to 18 March 2020 using the terms and operators (“caffeine” OR “energy drinks” OR “beverage”) AND (“cognitive”) AND (“sport” OR “exercise”). 

### 2.3. Eligibility Criteria

Records were identified according to the inclusion criteria: (1) reports with clear information regarding the administration of caffeine (relative dose of caffeine per kg of body weight and/or an absolute dose of caffeine with information about body weight, timing of caffeine intake before the onset of performance tests, etc.); (2) caffeine administered in the form of a beverage, coffee, energy bar, gum, or capsule; (3) studies including a placebo group; (4) experiment is well-designed and involves the ingestion of a dose of caffeine or a caffeine-containing product before and/or during sport or exercise; (5) design is a double-blind, randomized cross-over experiment; and (6) article language is English. 

The following studies were excluded: (1) those conducted in ill or injured participants; (2) those in which participants were not adults; (3) those in which caffeine doses below 1 mg/kg or above 9 mg/kg were used; (4) those lacking a true placebo condition; and (5) those lacking pre-experimental standardization, such as the elimination of dietary sources of caffeine 24 h before testing. To increase the power of the analysis, no filters were applied to the athletes’ training status or sex.

Once the records were identified, duplicates were first removed. Next, based on the titles and abstracts of articles, all of those that did not meet the eligibility criteria were excluded. Finally, the full texts of the resulting articles were read, and those that did not meet the inclusion criteria were removed.

The information extracted from the records identified was: study source (including authors and year of publication), study design, caffeine administration (dose and timing), sample size, characteristics of the participants (training status and sex), and outcomes of the interventions.

For the meta-analysis, studies were excluded if they did not report adequate performance data (i.e., no mean ± standard deviation (SD) or appropriate effect sizes). If a study contained multiple intervention arms (e.g., involving different doses of caffeine, dosing regimens, or participant populations), more than one of which was eligible for inclusion, the separate arms were treated as discrete studies, termed trials. As single trials often measured performance multiple times and/or used multiple tests that generated several different outcomes, each trial could provide more than one effect estimate (EE).

### 2.4. Quality Assessment

To determine the methodological quality of the quantitative studies reviewed, the criteria of Law et al. were used [42]. According to these criteria, the following factors were assessed as 16 items: purpose, literature background, design, sample, outcomes, intervention, results, dropouts, conclusions, and implications. Each of these items was awarded a score of 1 (meets the criterion), 0 (does not meet the criterion), or NA (not applicable). The score of all 16 items together was the measure of the methodological quality of the study. Studies awarded a score of 12 to 15 were considered to be of good to excellent methodological quality [42]. 

### 2.5. Performance Outcomes

All objective and subjective measurements of cognitive performance were considered in the review (see Table 2), while only objective measurements were included in the meta-analysis. 

The different cognitive performance tests assessed the different functions listed in Table 3. Within each domain, response speed and response accuracy data were handled separately.

### 2.6. Data Synthesis and Meta-Analysis

For each performance outcome, independent-group Hedges’ g intervention EEs [43] were calculated by standardizing the mean difference between the control and intervention performance scores against the pooled SD and correcting for bias due to a small sample size. The magnitude of the effect was as defined by Cohen [44]: Hedges’ g ≤ 0.2 = small, 0.2–0.5 = medium, and ≥ 0.8 = large, whereby a positive value indicated a beneficial effect of caffeine, irrespective of the performance outcome measured. If a trial repeated the same performance test two (or more) times within a 6 h period, and no additional caffeine was provided between tests, the resulting Hedges’ g values were averaged into a single EE (with the sample size increased accordingly). Meta-analyses were performed to determine the effect of caffeine on: (1) attention (response speed and accuracy); (2) simple reaction time (response speed); (3) choice reaction time (response speed and accuracy); and (4) inhibitory control (response speed and accuracy). Remaining performance outcomes (i.e., memory and detection) were unsuitable for meta-analysis, either because the data were derived from too small a number of studies or performance tests and outcomes were too heterogeneous for a meaningful meta-analysis. 

When fixed-effect models were not suitable for the studies, weighted mean treatment effects were calculated using random-effect models, whereby trials were weighted by the inverse variance of the performance change. Significance was considered if the 95% confidence interval (CI) did not include zero. Heterogeneity was assessed using Cochran’s Q and $I^{2}$ index. For Cochran’s Q, a *p*-value < 0.10 was used to indicate significant heterogeneity. Low, moderate, and high heterogeneity was indicated by an $I^{2}$ value of 25, 50, and 75%, respectively [45]. All statistical tests were performed using the software packages IBM SPSS version 25.0 and Review Manager version 5.3. All data are provided as mean ± SD.

## 3. Results

### 3.1. Selection of Studies

**Figure 1:**
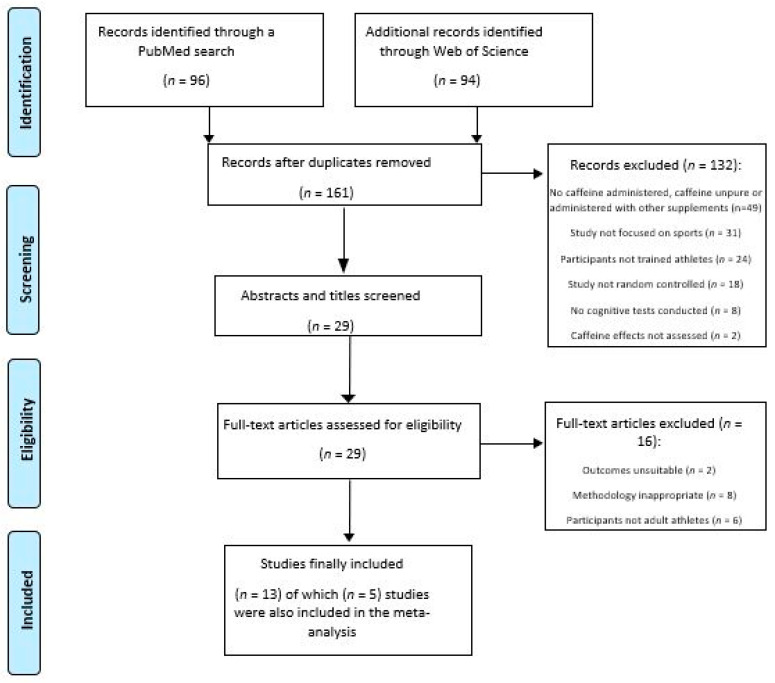
Selection of studies.

In the database search, 190 records were identified. Of these, 29 duplicates were removed and 132 articles were excluded after their titles and abstracts had been screened for eligibility. This left 29 studies for which the full-text articles were screened. After removing a further 16 reports according to our inclusion/exclusion criteria, 13 studies remained for review and meta-analysis (see Figure 1).

### 3.2. Quality Assessment of Studies

Scores awarded to the 13 reviewed studies are provided in Table 4. According to these scores, the methodological quality of one study (8%) was classified as excellent [38], of nine studies (69%) as very good [33,37,39,46,47,48,49,50,51], and of three studies (23%) as good [52,53,54]. 

### 3.3. Caffeine Supplementation

Participants were adult athletes of both genders (158 men and 36 women) who took part in professional/elite (*n* = 42), semi-professional (*n* = 14), or amateur (*n* = 109) sports activities. In one study, participants were ten athletes from recreational to professional levels without specifying training status in detail. Of the 13 studies reviewed, six included female athletes. In five of the 13 studies, participants were team sport players (*n* = 80).

In seven of the 13 studies reviewed, caffeine was administered based on the subject’s body weight, while an absolute dose was provided for the participants of six studies. The caffeine dose employed was less than 3 mg/kg in four studies, 3 mg/kg to 6 mg/kg in eight, and different doses (2 and 4 mg/kg) in one. 

The caffeine administration mode was capsules in five studies, energy drinks in three, a 68.8 mg/l carbohydrate solution in one, chewing gum in one, mouth wash in one, tablets in one, and a 45 g carbohydrate energy bar in the final one.

Most investigations administered caffeine 30–60 min prior to testing (*n* = 8). In the studies conducted by Cesareo et al. [50] and Hogervorst et al. [37], caffeine was ingested 1.5 h before the start of exercise. Furthermore, in four studies, besides administrating caffeine before the start of exercise, the supplement was also taken during exercise [37,38,49,52]. Hogervorst et al. [37] used a protocol that included the ingestion of caffeine 1 h before the test and every 20 min during the protocol. Mumford et al. [48] administered caffeine 120 min after starting a game of golf. Finally, Russell et al. [52] employed caffeine 15 min during exercise through the use of caffeinated gums. In summary, the different studies examined the effect of acute caffeine supplementation taken from 5–60 min prior to testing on cognitive performance during sports activity.

### 3.4. Outcome Measures

Table 5 and Table 6 provide information about the studies reviewed: author/s, year of publication, and participants and their characteristics; the study design, including the control group; the supplementation mode, dose, and timing; the outcomes analyzed or the main effects on cognitive performance measured using objective tools (*n* = 10; Table 5) or self-reports (*n* = 6; Table 6); and finally, the results or main conclusions.

### 3.5. Meta-analysis.

Of the 13 studies reviewed, eight could not be included in the meta-analysis because: only one trial was reported in [47,49,51,53]; no cognitive tasks were performed and only mood was tested in [48,50,54]; and means and standard deviations were not provided in [38]. This left five studies that fulfilled the criteria for meta-analysis. These studies consisted of 18 trials (*n* = 988 participants).

**Figure 2:**
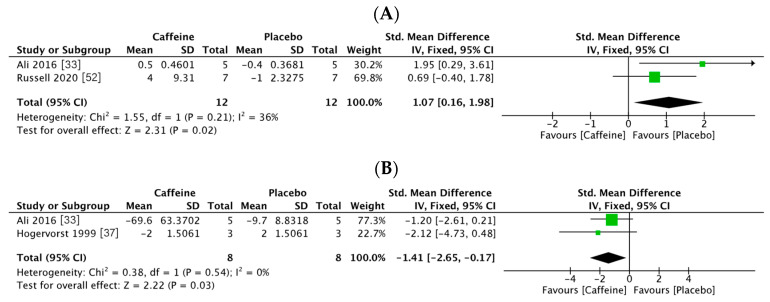
Forest plots of attention performance observed in the athletes in conditions of supplementation with caffeine vs. a placebo. (**A**) Response accuracy; (**B**) response speed.

Only two trials described the accuracy of attention (24 participants: 12 in the caffeine group and 12 in the placebo group) [48,52]. A fixed effects model was used to analyze response accuracy according to the heterogeneity of the data ($I^{2}$ = 36%, *p* = 0.02). After pooling the data, the caffeine group showed a significantly greater accuracy of attention than the placebo group (standard mean difference SMD = 1.07, 95% CI: 0.16, 1.98, *p* = 0.02, Figure 2A). In two trials, the response speed was measured (25 participants: eight in the caffeine group and eight in the placebo group) [38,48]. Again, a fixed effects model was used because of the heterogeneity of the data ($I^{2}$ = 0%, *p* = 0.45). Pooled analysis revealed a significantly improved response accuracy in the caffeine group (SMD = −1.41, 95% CI: −2.65, −0.17, *p* = 0.03, Figure 2B).

**Figure 3:**
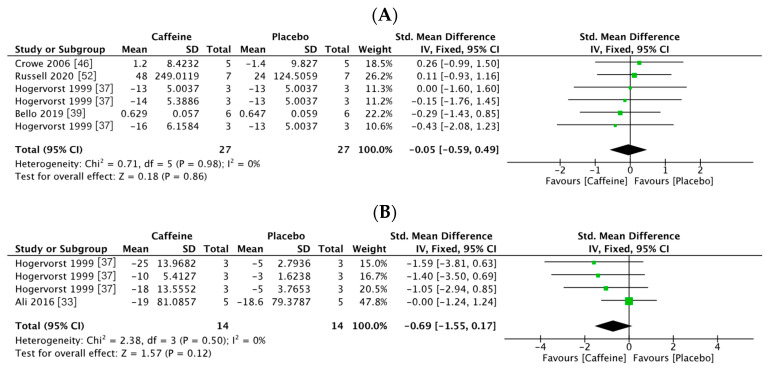
Forest plots of reaction times observed in the athletes in conditions of supplementation with caffeine vs. a placebo. (**A**) Simple reaction time; (**B**) choice reaction time.

Of the 18 trials, six recorded the simple reaction time (54 participants: 27 in the caffeine group and 27 in the placebo group) [37,39,46,52]. Because of the heterogeneity of the test result ($I^{2}$ = 0%, *p* = 0.98), a fixed effects model was used. Results indicated no significant difference in simple reaction time between the caffeine and placebo groups (SMD = −0.05, 95% CI: −0.59, 0.49, *p* = 0.86, Figure 3A). Four trials reported on the choice reaction time (28 participants: 14 in the caffeine group and 14 in the placebo group) [33,37]. A fixed effects model was used to analyze the data ($I^{2}$ = 0%, *p* =0.5). Results revealed no significant difference in choice reaction time between the groups (SMD = −0.69, 95% CI: −1.15, 0.17, *p* = 0.12, Figure 3B).

**Figure 4:**
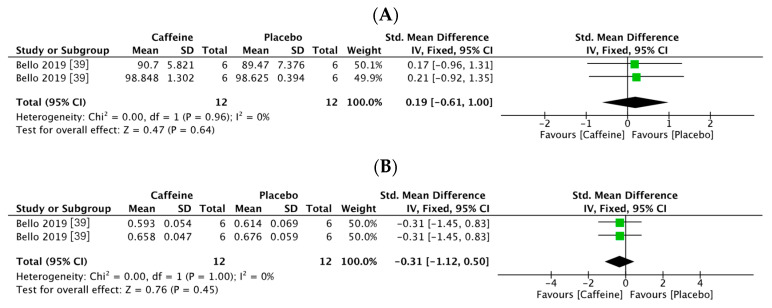
Forest plots of inhibitory control responses observed in athletes in conditions of supplementation with caffeine vs. a placebo. (**A**) Response accuracy; (**B**) response speed.

Two trials tested accuracy in inhibitory control (24 participants: 12 in the caffeine group and 12 in the placebo group) [39]. According to the heterogeneity of the test result ($I^{2}$ = 0%, *p* = 0.96), a fixed effects model was used to analyze the data. Results indicated no significant difference in response accuracy for inhibitory control between the groups (SMD = 0.19, 95% CI: −0.61, 1.00, *p* = 0.64, Figure 4A). In addition, two trials provided response speeds (24 participants: 12 in the caffeine group and 12 in the placebo group) [39]. A random effects model was used to analyze the data according to the heterogeneity of the test result (=0%, *p* = 1.00). Results indicated no significant difference in response speed between the groups (SMD = −0.31, 95% CI: −1.12, 0.50, *p* = 0.45, Figure 4B).

## 4. Discussion

The purpose of this systematic review and meta-analysis was to summarize all of the scientific evidence available regarding the effects of acute caffeine supplementation on variables related to cognitive performance in sport. Due to the different outcome measures of the studies, the variables described below were clustered for more comprehensive scrutiny.

### 4.1. Objective Measurements of Cognitive Performance 

Ten of the 13 studies reviewed here used objective measurements to examine the effects of caffeine on cognitive performance. Five of these 10 studies were included in the meta-analysis. Overall, the results indicated that caffeine improves attention performance (relative to a placebo) in athletes taking caffeine supplements before the start of their routine training or sports exercise. However, reaction time and inhibitory control were not improved according to the results of our meta-analysis. Thus, the ingestion of caffeine appears to be an effective measure to enhance an athlete’s attention when training or participating in their given sports activity. 

Although the data available for meta-analysis were insufficient, these investigations showed an improvement in response to caffeine intake across a variety of cognitive domains. Indeed, many studies have revealed that caffeine may benefit relatively higher-order processes, such as visual selective attention [55,56]. In a study by Hogervorst et al. [37], 15 male professional endurance athletes (age: 23.3 ± 3.6 years) were administered low caffeine doses (≈1.2, 1.8, and 2.56 mg/kg) 60 min before exercise and given the same solution every 20 min during exercise. These authors observed a faster Stroop test time, indicating improved visual selective attention in their caffeine group compared to the intake of the placebo after exercise. This effect was also noted in a subsequent study in endurance-trained men [38], in which the intake of 100 mg (≈1.36 mg/kg) of caffeine in a 45 g carbohydrate energy bar significantly improved reaction time in the Stroop test, with no impacts on the number of correct answers in this test. These effects of caffeine supplementation on the Stroop test observed in endurance athletes [37,38] could be of a lower magnitude in team sport athletes. In effect, in the study reviewed here conducted in female team game players, a trend towards significant differences was detected in the interaction treatment × time for a faster reaction time (*p* = 0.070) and higher percentage of correct responses in the Stroop test (*p* = 0.072) after caffeine supplementation compared to the placebo [33]. In contrast, Russell et al. detected no difference in response congruence in the Stroop test in professional male rugby players supplemented with caffeine (4.1 ± 0.5 mg/kg) or a placebo [52]. These results suggest that the impacts of caffeine on the Stroop test could depend on the sport’s modality, with more pronounced effects of caffeine supplementation produced in modalities with lower attention demands, such as endurance compared to team sports.

The flanker task also tests the processing of cognitive function underlying visual attention. In an investigation that analyzed the effect of caffeine supplementation in the flanker task [51], the intake of 5 mg/kg of caffeine 60 min before anaerobic exercise enhanced the response speed in both the congruent and incongruent condition, whereas, for accuracy, there was no significant substance × time interaction for these two conditions. Taken together, these results indicate that caffeine supplementation might be a practical way to improve the speed of visual selective attention, besides increasing accuracy. The tests, dependent variables, and cognitive functions assessed in the studies reviewed here are described in Table 3. Attention shifting was also tested in other studies reviewed here. According to Hogervorst et al. [37], a low caffeine dose can improve signal detection speed and accuracy. In another investigation, Hogervorst’s group [38] also found that an energy bar containing 100 mg (≈1.36 mg/kg) of caffeine and 45 g of carbohydrates, given before and during exercise, can enhance attention shifting. Together, these two investigations suggest that an acute dose of caffeine before and during exercise can improve attention in sports. 

Share et al. [53] assessed whether cognitive functions in clay target shooting, such as reaction time and target tracking time, were affected by two different doses of caffeine supplements (2 mg/kg and 4 mg/kg) compared with a placebo. In that study, no effect of caffeine supplementation was detected. Furthermore, Church et al. [47] observed no difference in the ability to track multiple objects in recreationally active male subjects in response to a caffeine supplement (3 mg/kg) ingested 60 min before exercise. Thus, it is likely that caffeine does not influence tracking ability in sports. In contrast, there is also evidence to suggest that, whereas caffeine may improve overall processing speed in tasks requiring higher-order functions, these improvements cannot be attributed to specific effects on selective visual attention [57].

The results of some of the reviewed studies suggest that caffeine reduces response times and error rates in tasks such as simple reaction time [58] and choice reaction time [59,60]. Our meta-analysis, however, revealed that this effect may not translate to the sports area. Many studies have used the simple reaction time test (SRT) to determine cognitive performance in sports research. Crowe et al. [46] used the simple visual reaction time test. Their main finding was that caffeine was not able to speed up cognitive reaction time, perhaps because the stimulant was consumed early (90 min before starting) in relation to peak plasma levels (≈60 min after supplementation) [15], and thus the effects of caffeine would have been limited due to incorrect timing. Similarly, Bello et al. [39] found that the intake of 275 mg (≈3.69 mg/kg) of caffeine in capsules 30 min prior to exercise in male and female professional soccer players improved simple psychomotor speed performance. Chewing gum containing caffeine is considered a better way to absorb the stimulant rapidly than capsules and drinks [61]. Russell et al. [52] analyzed the effect of chewing caffeinated gum (4.1 ± 0.5 mg/kg caffeine) every 15 min during exercise in professional male academy rugby players, but no interaction treatment × time was observed in simple reaction time. Church et al. [47] and Share et al. [53] used non-standardized measures to test reaction time. These authors also separately tested upper body and lower body reaction time, and no differences emerged between caffeine and the placebo in both studies [47,53]. Hogervorst et al. [37] used the motor choice reaction time test (MCRT) to test complex decision speed and complex response preparation speed. They found that, after exercise, both complex psychomotor speed and S-R incompatible choice speed were significantly faster after the intake of low dosage caffeine than the placebo. Furthermore, no effects of caffeine supplementation (6 mg/kg 60 min prior to exercise) were observed by Ali et al. [33] on the choice reaction time (CRT), a test measuring complex decision-making capacity. Bello et al. [39], in a study performed in professional soccer players, examined the effect of 275 mg of caffeine (≈3.69 mg/kg) ingested 30 min before exercise on the results of the go/no-go test, which assesses cognitive performance in sports. In this study conducted in 2019 [39], Bello et al. described significant effects of acute caffeine supplementation on SRT, CRT, and cognitive load reaction time (COGRT), suggesting that caffeine supplementation might improve choice reaction time in sports.

We were unable to find any evidence that caffeine improves inhibitory control, according to our athletes’ response speed or accuracy after exercise. Some authors argue that the benefits of caffeine for executive control are only reliably seen with relatively high doses of caffeine in individuals with low-consumption profiles [62]. These effects may be specific to reactive rather than active inhibition [63]. However, while there is some evidence that reactive inhibition may be improved as a result of caffeine consumption [64,65], no study examining active inhibition has detected such effects [66]. Bello et al. [39] reported that a moderate-dose (275 mg ≈3.69 mg/kg) caffeine capsule administered to professional soccer players 30 min before exercise was unable to improve inhibitory control.

Memory is another cognitive function tested in many investigations. Hogervorst et al. [37,38] used the word learning test to explore the effects of caffeine supplementation in sports on long-term memory. The results of these studies showed that, after exercise, word learning speed improved with caffeine, but word recall was unaffected. However, Crowe et al. [46] found no significant short-term memory changes after caffeine supplementation compared to the placebo [46]. These results contrast with the findings of Hogervorst et al. [38] relative to the rapid visual information processing task in that, after exercise, reaction and the miss rate decreased significantly, and the true positive rate increased significantly in response to caffeine. Pomportes et al. [49] examined the effect of an alternative mode of caffeine administration (mouthwash) in male and female recreational cyclists who rinsed their mouths with a solution containing 67 mg/25 mL (≈0.93 mg/kg) of caffeine immediately before a submaximal cycling test and every 13 min during the test. The cyclists experienced decreased memory over time, but with a lower variance observed in the caffeine supplementation than the placebo condition, suggesting that low-dose caffeine improves memory-related mechanism performance in sport. Collectively, these results suggest that long-term memory is only affected under strict conditions, such as the intake of low-dose caffeine immediately before and every 20 min during exercise in male professional cyclists or triathletes. 

### 4.2. Self-Reported Subjective Scales

Six of the studies reviewed used subjective scales to assess mood state in conditions of caffeine supplementation and exercise (Table 5). The VAS-F scale has been widely used for different research purposes. Church et al. [47] noted significant condition and time interaction in self-reported energy levels and acute caffeine intake, while no such interaction was observed in alertness and focus. According to a study by Mumford et al. [48], the consumption of a caffeine drink containing 155 mg (≈1.9 mg/kg) of caffeine had no effects on alertness, concentration, and overall confidence in male recreational golfers. Furthermore, there was a significant condition and time interaction effect on self-perceived ratings of fatigue, such that fatigue increased only in the placebo condition. In another study, Cesareo et al. [50] observed a significant caffeine effect on energy, focus, and motivation to exercise from baseline to 90 min post-treatment after 300 mg (≈3.6 mg/kg) of caffeine supplementation in resistance-trained athletes. Unlike fatigue, mean differences in energy, focus, and motivation to exercise were significantly higher in the caffeine trials compared to the placebo, suggesting that the intake of caffeine might increase the feeling of energy in sports. These results are in accordance with the findings of Ali et al. [33], who reported an interaction effect with scores increasing in the caffeine trial and decreasing in the placebo trial over time on ratings of vigor. These authors also detected a trend towards lower fatigue scores in their caffeine trial compared with the placebo trial, and lower ratings over time. The feeling scale (FS) and felt arousal scale (FAS) were used to measure feelings and arousal. Ratings of pleasure and arousal were higher after caffeine supplementation compared to a placebo. Taken together, these data suggest that caffeine in the form of drinks or capsules can affect a person’s mood state, especially in terms of feeling more energetic (vigor), pleasure, and arousal (see self-reported scores in Table 3).

For ratings of perceived exertion (RPE), Foskett et al. [54] conducted a study designed to examine the effects of caffeine (6 mg/kg, 60 min at the start of exercise) on perceptual measures during a simulated team sport in male professional soccer players. No differences were observed between trials, although perceptions of effort increased with exercise duration in both trials. In contrast, Duncan et al. [51] found that RPE values were significantly lower under conditions of caffeine than the placebo. 

### 4.3. Strengths, Limitations, and Future Lines of Research

Our review and meta-analysis have several limitations related to the experimental design and the different research protocols and cognitive performance tests used in the studies reviewed. Although we selected studies comparing the effects of caffeine supplementation to a similar placebo condition in a double-blind design, in some studies, caffeine was taken with other compounds, such as carbohydrates. Thus, it could be that synergistic or antagonistic effects were produced on physical and cognitive performance. In addition, the different sources of caffeine (capsules, tablets, gums, mouthwash, drinks, and energy bars) could affect caffeine pharmacokinetics [17] and, consequently, the results of the different studies. The different doses and regimens could also have affected some of the outcome measures. Moreover, the different training levels of the athletes could modulate the effects of caffeine [67]. However, the low number of studies prevented us from identifying different effects of caffeine supplementation on cognitive functions according to participant competition level. Another limitation was that, based on the studies included in the systematic review and meta-analysis, it was not possible to detect an effect of sex on the ergogenicity of caffeine [16]. Despite these limitations, our findings point to an ergogenic effect of caffeine in improving participants’ attention, energy, and mood, and no or little effect on simple reaction time, choice reaction time, memory, or fatigue. As cognitive function is a complex mechanism that includes various mental operations, the measures investigated in this systematic review only represent a small proportion of these factors. Further work is needed to confirm the impacts of caffeine on more standardized measures of cognitive function and elucidate whether these effects vary according to factors such as an individual’s training status, sex, or age.

## 5. Conclusions

In summary, the intake of an acute low/moderate dose of caffeine before and/or during exercise can improve cognitive functions, such as attention, along with energy and mood. It can also improve simple reaction time, choice reaction time, memory, or fatigue, which may depend on the research protocols. So far, it has been shown that a single acute dose of caffeine has no detrimental effects on measures of some aspects of cognitive function during exercise. Moreover, acute caffeine supplementation affects neither target tracking nor multiple objects tracking, or ratings of perceived exertion during any form of exercise. Despite several benefits of caffeine on cognitive performance in sports suggested by this review, the use of caffeine supplementation still needs to be assessed for side effects typically associated with the consumption of this stimulant.

## Figures and Tables

**Table 1:** PICOS criteria for the inclusion of studies in the systematic review.

Parameter	Inclusion Criteria
Population	Adult athletes
Intervention	Caffeine supplementation
Comparators	Placebo supplementation
Outcomes	Variables related to cognitive performance in sports, including reaction time, memory, focus, concentration, alertness, fatigue, motivation, and attention
Study design	Double-blind/single-blind and randomized cross-over design

**Table 2:** Subjective scales and score systems used to measure cognitive functions.

Scale	Score System	Cognitive Function
Visual analogue scale for fatigue (VAS-F)	Likert-type scale	Fatigue Energy Alertness Focus
Profile of mood states (POMS)	5-point scale	Fatigue Anger Vigor Tension Esteem Confusion Depression
Felling scale (FS)	11-point scale	Pleasure/Displeasure
Felt arousal scale (FAS)	6-point scale	Perceived arousal
Rating of perceived exertion (RPE)	6–20 point scale	How hard you are working

**Table 3:** Objective tests, dependent variables, and cognitive functions measured.

	Test	Dependent Variables	Function Measured
Attention	SCWT (Stroop Color and Word Test)	Time to read card (s) Congruent accuracy (%) Incongruent accuracy (%)	Visual selective attention Attention bias Sensitivity to interference Ability to suppress an automated response
	Flanker test	RT (ms) Congruent accuracy (%) Incongruent accuracy (%)	Visual selective attention Ability to manage interference
	RVIP (rapid visual information processing task)	RT (ms) True positive (TP) rate (%) Miss rate (%)	Visual selective attention Working memory
	Visual search test	RT for correct responses (ms) Accuracy (%)	Attention shifting
	SDT (signal detection task)	RT (ms) Efficiency (%) Efficiency of visual signal detection (A’)	Speed of signal detection Efficiency of signal detection/attention shifting
Reaction Time	Simple visual reaction time test	RT (ms)	Simple psychomotor speed
	MCRT (motor choice reaction time test)	Simple RT (ms) Choice RT (ms) S-R incompatible choice RT (ms)	Simple psychomotor speed Complex decision speed Complex response preparation speed
	Choice reaction time (CRT) test	Choice RT (ms)	Decision-making time
Inhibitory Control	Go/no-go task	Choice RT (ms) Choice RT score (%)	Complex decision speed and accuracy Inhibitory control
	Go/no-go and cognitive load task	Cognitive load RT (ms) Cognitive load RT score (%)	Inhibitory control
	Simon task	Congruent RT Incongruent RT Errors rate (%)	The ability to inhibit pre-potent responses
Memory	VVLT (Visual Verbal Learning Test)	RT (ms) Recognition of words (0~15)	Speed of retrieval from long-term memory Storage in long-term memory
	Number recall test	Percentage number recall	Short-term memory
Internal Time-Keeping Mechanisms	Duration production task	Produced duration (ms) Variance (ms)	Effect of changes in the speed of internal time-keeping mechanisms

**Table 4:** Methodological quality of the studies (*n* = 13).

Item	1	2	3	4	5	6	7	8	9	10	11	12	13	14	15	16	Total	MQ
Study																		
Ali et al. [33]	1	1	1	1	1	1	1	1	1	1	0	1	1	0	1	1	14	VG
Hogervorst et al. [37]	1	1	1	1	0	1	1	1	1	1	0	1	1	0	1	1	13	VG
Hogervorst et al. [38]	1	1	1	1	1	1	1	1	1	1	1	1	1	0	1	1	15	E
Bello et al. [39]	1	1	1	1	0	1	1	1	1	1	0	1	1	1	1	1	14	VG
Crowe et al. [46]	1	1	1	1	0	1	1	1	1	1	1	1	1	0	1	1	14	VG
Church et al. [47]	1	1	1	1	0	1	1	1	1	1	1	1	1	0	1	1	14	VG
Mumford et al. [48]	1	1	1	1	0	1	1	1	1	1	1	1	1	0	1	1	14	VG
Pomportes et al. [49]	1	1	1	1	0	1	1	1	1	1	1	1	1	0	1	1	14	VG
Cesareo et al. [50]	1	1	1	1	0	1	1	1	1	1	1	1	1	0	1	1	14	VG
Duncan et al. [51]	1	1	1	1	0	1	1	1	1	1	0	1	1	0	1	1	13	VG
Russell et al. [52]	1	1	1	1	0	1	1	1	1	1	0	1	1	0	1	0	12	G
Share et al. [53]	1	1	1	1	0	1	0	0	1	1	1	1	1	0	1	1	12	G
Foskett et al. [54]	1	1	1	1	0	1	1	1	1	0	0	1	1	0	1	1	12	G

Item score: 1 = criterion fulfilled; 0 = criterion not fulfilled. MQ: methodological quality (MQ); G: good (11–12 points); VG: very good (13–14 points); E: excellent (15 points).

**Table 5:** Details and results of the studies reviewed investigating the effect of acute caffeine supplementation compared to a placebo on objective measures of cognitive performance.

Study	Population	Intervention	Outcomes Analyzed	Main Results vs. Placebo
Russell et al. [52]	14 male professional academy rugby players (18 ± 1 years)	4.1 ± 0.5 mg/kg of caffeine (gum) 15 min before and during exercise	SRT test Stroop test	SRT Congruent response accuracy Incongruent response accuracy
Duncan et al. [51]	12 male subjects accustomed to regular high-intensity exercise (21.4 ± 4.4 years)	5 mg/kg of caffeine (capsules) 60 min before start	Modified flanker task	↓ Congruent RT ↓ Incongruent RT Congruent response accuracy Incongruent response accuracy
Bello et al. [39]	12 male (21.8 ± 2.53) and 15 female (19.65 ± 3.62) professional soccer players	275 mg (≈3.69 mg/kg) caffeine capsule 30 min before start	SRT test Go/no-go Go/no-go and COGRT wrong	↑ SRT CRT CRT score COGRT COGRT score COGRT wrong answer
Pomportes et al. [49]	16 male and six female recreational cyclists (26 ± 8) years	67 mg/25 mL (≈0.93 mg/kg) of caffeine + 7% carbohydrate mouthwash Immediately before start and every 13 min during exercise	Duration-production task Simon task	↓ Produced duration ↓ Variance ↑ Congruent mean RT ↓ Incongruent mean RT Error rate (%)
Ali et al. [33]	10 female team game players from recreational to international (24 ± 4 years)	6 mg/kg caffeine capsule 60 min before start	CRT test Stroop test	CRT RT CRT accuracy ‡ Stroop test RT † Stroop test accuracy
Church et al. [47]	10 male recreationally active subjects (25.5 ± 1.8 years)	3 mg/kg caffeine drink 60 min before start	Reaction time test Multiple object tracking	Upper body RT Lower body RT Multiple object tracking
Share et al. [53]	Seven male elite clay target shooters (28.4 ± 9.4 years)	2 or 4 mg/kg caffeine tablets 60 min before start	Reaction time test Tracking time of target	RT TT1 TT2
Hogervorst et al. [38]	24 well-trained male subjects (23 ± 5 years)	100 mg (≈1.36 mg/kg) of caffeine + 45 g carbohydrate energy bar Immediately before and every 55 min during exercise	Stroop Color and Word test RVIP test Visual search test Word learning Test	↓ Stroop RT Stroop accuracy ↓ RVIP RT ↑ RVIP PT rate ↓ RVIP miss rate ↓ Visual Search RT ↑ Visual Search accuracy Delayed recall words
Crowe et al. [46]	12 male and five female team sports players (21.1 ± 3.0 years)	6 mg/kg caffeine drink 90 min before start	Simple visual reaction time test Number recall test	RT Number recall
Hogervorst et al. [37]	15 male professional cyclists or triathletes (23.3 ± 3.6 years)	8 mL/kg of 150, 225, or 320 mg/l of caffeine + 68.8 mg/l carbohydrate solution (≈1.2, 1.8, or 2.56 mg/kg of caffeine) 60 min before start 3 mL/kg of 150, 225, or 320 mg/l of caffeine + 68.8 mg/l carbohydrate solution (≈0.45, 0.675, or 0.96 mg/kg of caffeine) every 20 min during exercise	Stroop Color and Word Test SDT test Motor choice reaction time test VVLT	↑ Stroop speed ↑ SDT efficiency ↑ SDT speed ↑ Simple psychomotor speed ↑ Complex psychomotor speed ↑ S-R incompatible choice speed VVLT delayed number recall ↑ VVLT speed

↑ Statistically significant increase; ↓ statistically significant decrease; † increasing tendency; ‡ decreasing tendency. Without any marks indicates that there were no differences between caffeine and the placebo for the measures. RT: reaction time; SRT: simple reaction time; CRT: choice reaction time; COGRT: cognitive load reaction time; RVIP: rapid visual information processing; SDT: signal detection test; VVLT: visual verbal learning test.

**Table 6:** Details and results of the studies reviewed investigating the effect of acute caffeine supplementation compared to a placebo on self-reported measures of cognitive performance.

Study	Population	Intervention	Outcomes Analyzed	Main Conclusion
Duncan et al. [51]	12 male subjects accustomed to regular high-intensity exercise (21.4 ± 4.4 years)	5 mg/kg of caffeine (capsules) 60 min before start	RPE VAS	↓ RPE RTIPE RTIME
Cesareo et al. [50]	12 male resistance-trained subjects (23.2 ± 3.1)	300 mg (≈3.6 mg/kg) caffeine capsule 90 min before start	VAS-F	↑ Energy ↑ Focus ↑ Motivation to exercise Fatigue
Ali et al. [33]	10 female team game players from recreational to international (24 ± 4 years)	6 mg/kg caffeine capsule 60 min before start	FS FAS POMS	↑ Rating of pleasure ↑ Rating of arousal ↑ Rating of vigor ‡ Rating of fatigue
Mumford et al. [48]	12 male recreational golfers (34.8 ± 13.9 years)	155 mg (≈1.9 mg/kg) caffeine drink 25~35 min before start and 120 min during exercise	VAS-F	Alertness Overall confidence Concentration ↑ Energy ↑ Fatigue
Church et al. [47]	10 male recreationally active subjects (25.5 ± 1.8 years)	3 mg/kg caffeine drink 60 min before start	VAS-F	↑ Energy Alertness Focus
Foskett et al. [54]	12 male professional soccer players (23.8 ± 4.5 years)	6 mg/kg caffeine capsules 60 min before start	RPE	RPE

↑ Statistically significant increase; ↓ statistically significant decrease; ‡ tendency for decreasing. Without any marks indicates that there were no differences between caffeine and the placebo for those measures. RPE: ratings of perceived exertion; VAS-F: visual analogue scale for fatigue; RTIPE: readiness to invest physical effort; RTIME: readiness to invest mental effort; FS: feeling scale; FAS: felt arousal scale; POMS: profile of mood states.

## Data Availability

The datasets used and/or analyzed during the current study are available from the corresponding author on reasonable request.
